# MAIT Cells in Liver Disease

**DOI:** 10.3390/cells15010069

**Published:** 2025-12-31

**Authors:** Adiba I. Azad, Florencia Gutierrez, Gregory J. Gores

**Affiliations:** Division of Gastroenterology and Hepatology, Mayo Clinic, Rochester, MN 55905, USA; gutierrez.florencia@mayo.edu

**Keywords:** mucosal-associated invariant T (MAIT) cells, chronic liver disease, unconventional T cells, liver fibrosis, viral hepatitis, primary sclerosing cholangitis, metabolic dysfunction-associated steatotic liver disease, alcohol-associated liver disease, liver fibrosis

## Abstract

Mucosal-associated invariant T (MAIT) cells are abundant innate-like T lymphocytes in the human liver which can provide antimicrobial defense, amplify inflammatory processes and mediate tissue repair and fibrosis depending on microenvironmental cues. Chronic liver diseases of diverse etiologies, including viral hepatitis, metabolic dysfunction-associated steatotic liver disease, alcohol-associated liver disease, biliary tract disease, autoimmune hepatitis and hepatocellular carcinoma are accompanied by numerical and functional adjustments in the MAIT cell population. In this review, we integrate existing data on MAIT cell markers and functions in diverse liver diseases, comparing how these cells are similarly or differentially shaped by distinct pathogenic contexts. Finally, we propose a spatially anchored conceptual and technical framework to study MAIT cell biology in liver disease.

## 1. Introduction

**Figure 1:**
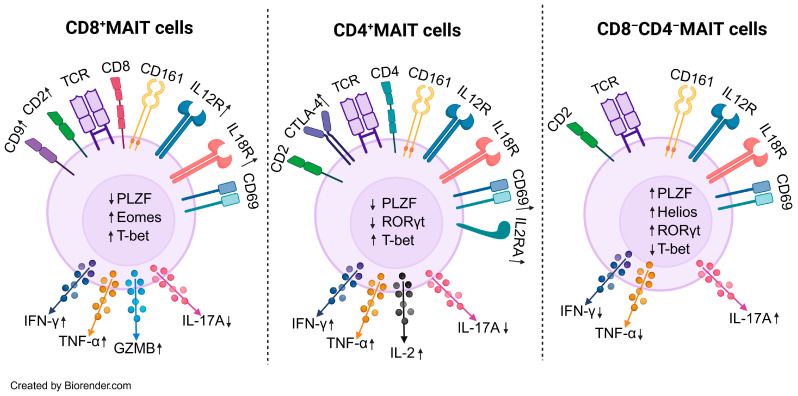
MAIT cell subset immunophenotype. CD8^+^ MAIT cells show higher expression of Eomes/T-bet and robust IFN-γ, TNF-α and GZMB production and reduced IL-17A. These markers are suggestive of a more cytotoxic phenotype. CD4^+^ MAIT cells show an enhanced expression of activation markers CD69, CTLA-4, and IL2. Unlike CD8^+^ MAIT cells they are not robust producers of cytotoxic molecules such as granzymes. CD8^−^CD4^−^ (double-negative) MAIT cells are enriched for PLZF, Helios and RORγt, and preferentially produce IL-17A with relatively reduced IFN-γ/TNF-α. Abbrv. IFN-γ, interferon gamma, TNF-α, tumor necrosis factor alpha, GZMB, granzyme B, PLZF, promyelocytic leukemia zinc finger, CTLA-4, cytotoxic T lymphocyte-associated protein 4, Eomes, eomesodermin. Long arrows denote cytokine secretion; adjacent ↑/↓ symbols indicate increased or decreased secretion.

There are two epithelial cell populations in the liver, hepatocytes and cholangiocytes. Hepatocytes receive blood flow directly from the portal circulation and can be considered a functional mucosa because of the unique composition of portal vein blood rich in nutritive and non-nutritive derivatives from the diet and microbes. The biliary tree is a branching network that begins as bile canaliculi between hepatocytes, which then coalesce to form progressively larger intrahepatic bile ducts that ultimately form extrahepatic bile ducts to drain bile from the liver into the gut. The surface of these bile ducts is lined by cholangiocytes which directly interact with and modify bile and therefore, represent a true mucosal surface. These features create a barrier-adjacent immune niche that must reconcile tolerance to routine intestinal and bile cargo with readiness to respond to infection or injury. In this context, the liver is strikingly enriched for innate-like lymphocytes, most notably mucosal-associated invariant T (MAIT) cells, which preferentially occupy sinusoidal (a vascular structure lined by fenestrated endothelial cells receiving portal blood) and peribiliary spaces (space adjacent to the cholangiocytes); display tissue-resident traits; and integrate microbial ligands with inflammatory cytokine cues. Indeed, a landmark study coined the term “mucosal-associated invariant T cells” after identifying this lineage in the intestinal mucosa and identifying MR1 as their antigen presenting molecule which is ubiquitously expressed [1]. MAIT cells belong to a population of “unconventional” T cells which also include gamma delta (gd) T cells and invariant natural killer (iNKT) cells. Together, the unconventional T cells break the mold of the classic adaptive paradigm where T lymphocytes recognize peptide antigens bound to polymorphic classical MHC molecules and, upon encounter, undergo antigen-specific clonal expansion and differentiation supported by an extraordinarily diverse T cell receptor (TCR) repertoire across the population. Instead, MAIT cells recognize a limited array of non-peptide metabolites presented on the monomorphic antigen presenting molecule MR1 [1]. While rare in the mouse (<1% of T cells), MAIT cells are enriched in the human, in which they represent 3–5% of peripheral blood, 10% of peripheral CD8^+^ T cells and 30–45% of liver T cells [2,3,4,5]. Human MAIT cells express an invariant TCRα chain comprising TRAV1-2 joined to TRAJ33 with a restricted TCRβ chain TRBV6 and TRBV20 [6]. The MAIT cell TCR recognizes a series of MR1-binding ligands which includes endogenous bile acids, drug molecules, folate, and riboflavin derivatives [7]. The most potent MR1-binding, MAIT-cell-activating ligands identified to date are 5-OP-RU and 5-OE-RU, intermediates in the riboflavin biosynthesis pathway, which are found across microbial species, but missing from the eukaryotic (human) host [8]. The most specific method of identifying MAIT cells is by using MR1-5-OP-RU tetramers in flow cytometry [8]. Like other T cell receptors, the MAIT TCR interaction with its cognate antigen presenting molecule MR1 and antigen (5-OP-RU) inherently demonstrates weak affinity and short half-life. Therefore, engineered fluorescently labeled MR1 molecules loaded with 5-OP-RU that are multimerized (i.e., MR1-5-OP-RU tetramers), are capable of engaging more than one TCR molecule on the MAIT cell surface and therefore, binds with enhanced avidity and dwell time. These features of “MR1 tetramers” enable the identification of MAIT cells even when they are rare populations, with high specificity. Circulating MAIT cells from healthy donors can be identified using surrogate set of markers TCRVα7.2^+^ CD161^+^ (and/or CD26^+^). Surrogate markers, especially CD161 may lead to the inconsistent identification of MAIT cells, as CD161 can be downregulated in activation, and is also expressed by other cells [9,10,11]. Circulating MAIT cells in human blood are predominantly CD8^+^ (80%), CD8^−^ CD4^−^ (20%) and a small minority being CD4^+^ [2]. CD8^+^ MAIT cells numerically dominate the circulating and intrahepatic MAIT population and display strong cytotoxic characteristics; CD8^−^ CD4^−^ MAIT cells show increased IL-17 expression and a stronger propensity to activation induced cell death; and CD4^+^ MAIT cells expand in disease contexts and most recent evidence suggests this subtype can sense a broader range of antigens than classic CD8^+^ MAIT cells [12,13,14,15] (Figure 1).

The liver is exceptionally enriched in MAIT cells, which are attuned to microbial metabolites and to cues from stressed tissue. Beyond host defense where MAIT cells secrete cytokines and effector cytotoxic molecules, MAIT cells exhibit tissue repair phenotypes and crosstalk with parenchymal, myeloid, and stromal compartments eliciting a wound healing response. Fibrosis represents a pathologic iteration of wound healing and the shared final common pathway of chronic liver disease across etiologies including viral hepatitis, metabolic dysfunction-associated steatohepatitis (MASH), autoimmune and cholestatic disorders, biliary atresia, and primary liver cancer. Fibrogenesis unfolds within a state of chronic inflammation driven by cytokine and chemokine networks and the sustained recruitment and activation of immune cells. MAIT cells not only produce a broad array of cytokines and chemokines but also recruit and shape other immune populations while activating their own wound healing programs, positioning them to influence both inflammatory and reparative arms of the response to injury thereby promoting fibrosis. The convergence of MAIT cell abundance in the liver with these dual effector and tissue restorative functions makes it highly plausible that they govern trajectories from acute injury to chronic inflammation and fibrosis. This review is a synthesis of current knowledge on the geographic distribution, phenotype, and function of MAIT cells in liver pathologies, and concludes with addressing conceptual and technical gaps in MAIT cell biology in the context of liver disease. A summary of reported MAIT cell numerical and functional changes across these liver diseases is provided in Table 1.

## 2. Viral Hepatitis and MAIT Cells

Viral hepatitis B is a disease caused by the DNA hepatotropic hepatitis B virus (HBV). Viral hepatitis B comprise different phases, which are not necessarily sequential, and the phases reflect the interaction between HBV and the host’s immune response to HBV. HBeAg-positive chronic HBV infection phase is characterized by high HBV titers in the blood, but minimal to no liver inflammation and fibrosis. HBeAg-positive chronic hepatitis phase is characterized by high HBV titers and moderate to severe inflammation and rapid progression to fibrosis. HBeAg-negative chronic HBV infection phase is characterized by HBeAg-directed antibodies and low liver inflammation, and usually low HBV titers. HBeAg-negative chronic hepatitis phase is a severe form of the disease characterized by fluctuating HBV titers, and liver inflammation and fibrosis. Finally, acute liver failure from HBV acute infection is a life-threatening clinical syndrome which often requires liver transplantation for definitive therapy [16]. Across chronic hepatitis B (CHB) and HBV-related liver failure, MAIT cells represent a dynamically tuned compartment whose abundance, activation state, and transcriptional programs are generally dampened in disease. MAIT cell numbers decreased significantly in HBV blood and liver pointing to attrition and not redistribution of MAIT cells in disease. This was accompanied by a global decrease in cytokine production and increase in markers of chronic activation and exhaustion (PD-1, CD69, HLA-DR, CD38, CD39) [17,18,19,20]. In treatment-naïve CHB, MAIT cells were not numerically depleted in either blood or liver compared with healthy controls but were hyperactivated with increased expression of elevated CD38 that declined on nucleos(t)ide analog therapy (entecavir) and a shift toward heightened granzyme B production after *E. coli* stimulation. HBeAg-negative chronic hepatitis B patients who have a more aggressive infection with a higher risk of progression to cirrhosis, exhibited the lowest activation, suggesting that viral antigen load and/or inflammatory tone are principal drivers [21]. In contrast, another study described reduced peripheral MAIT frequencies in CHB accompanied by a checkpoint-rich, exhausted phenotype (PD-1, CTLA-4, TIM-3, CD57, HLA-DR/CD38) with impaired IFN-γ/TNF-α responses. PD-1 levels correlated positively with HBV DNA, linking MAIT dysfunction to ongoing viremia [17]. Similarly, it has been shown that CD8^+^ T cells adopt a more exhausted phenotype in patients with high HBV surface antigen (sAg) levels indicating the classical T cell response to chronic antigenic stimulation [22]. Therefore, it is highly conceivable MAIT cells would behave in a similar fashion. These apparently discordant frequency findings likely reflect differences in cohort composition (ethnicity, disease activity, HBeAg status and HBV viral DNA levels), sampling site (blood vs. paired liver), and technical factors (e.g., surrogate gating versus MR1-tetramer approaches and stimulation conditions). The studies were also descriptive without a functional assessment of the MAIT cells.

A mechanistic study queried whether MAIT cells have direct anti-viral properties against HBV and what factors (viral or non-viral) contribute to MAIT cell numbers and function in CHB. MAIT cell identity was simultaneously confirmed by MR1-5-OP-RU tetramer^+^ and Va7.2^+^CD161^+^ gating in blood and liver of HBV-infected and control patients. MAIT cells displayed MR1-dependent degranulation and cytotoxicity against HBV transfected hepatocytes [19]. MAIT cell numbers decreased significantly in HBV blood and liver suggesting true attrition, not compartmental redistribution. This was accompanied by a global decrease in cytokine production and an increase in the markers of chronic activation and exhaustion (PD-1, CD69, HLA-DR, CD38, CD39). The loss in MAIT cells was shown to be associated with the levels of direct bilirubin, independent of the fibrosis stage, with in vitro reduction in TCR-dependent MAIT expansion by direct bilirubin [19]. These studies more directly highlight MAIT cell activity in HBV infections and hepatitis.

Employing a high-resolution approach to study liver resident MAIT cells in CHB, Shao et al. used biopsy-based single-cell RNA-seq to delineate divergent intrahepatic MAIT states in histologic grades of CHB. They find an antiviral-skewed pro-inflammatory MAIT cluster enriched in earlier CHB grade vs. a more dysfunctional and quiescent MAIT cluster in a more advanced histologic stage [23]. In the context of acute HBV hepatitis, Xue et al. found profound MAIT depletion in HBV-related acute-on-chronic liver failure (lowest in liver failure > CHB > healthy), in parallel with elevated IL-12 and IL-18, implicating cytokine-driven activation/exhaustion in MAIT attrition; strikingly, circulating MAIT proportion outperformed the classic severity score of liver disease, i.e., the MELD score in predicting 90-day mortality, and partial numerical recovery was observed among survivors after treatment [24]. These data suggest MAIT cells as candidate biomarkers of disease severity and prognosis in HBV-associated liver failure. Key open questions include whether microbial translocation in CHB causes MAIT cell activation and subsequent exhaustion and attrition due to direct antigen supply or creating a milieu rich in cytokines.

MAIT cells show alterations in hepatitis D virus (HDV) coinfection, where the RNA virus HDV depends on HBV machinery to infect hepatocytes and hijacks the host hepatocyte proteins to replicate. In HDV coinfection, there is a more profound loss in MAIT cell numbers than in HBV-monoinfected individuals [20]. However, the functional impairment of MAIT cells in HDV coinfection is stimulus specific where TCR-dependent response is blunted but cytokine-driven response is preserved. The caveats of this study are the small number of subjects per group (<50) and limited liver biopsies available for in situ labeling. Hepatitis C virus (HCV) is a hepatotropic RNA virus that establishes chronic infection in the majority of exposed individuals, driving persistent hepatic inflammation that can progress over years to fibrosis, cirrhosis, and hepatocellular carcinoma (HCC). In HCV infection, MAIT cells show a characteristic triad of numerical loss, heightened activation/exhaustion, and stimulus-dependent functional impairment. Multiple cohorts report reduced circulating MAIT frequencies in chronic HCV [25,26,27] alongside blunted IFN-γ/TNF-α and altered degranulation that persists despite interferon-free DAA cure [28,29,30]. Within the liver, MAIT cells are likewise depleted in inflamed HCV tissue, and their activation tracks a myeloid cytokine milieu; intrahepatic analyses during DAA treatment highlight monocyte-derived IL-12/IL-18–driven activation rather than antigen-only effects [31]. Mechanistically, MAIT cells can be strongly activated by IL-18 (with IL-12) in a TCR-independent manner during human viral infections, helping to explain sustained activation/exhaustion signatures even when direct TCR ligation is limited [32]. Post-DAA recovery of the compartment is heterogeneous: some studies note the partial restoration of circulating MAIT numbers/function after cure [33], whereas others especially in HIV/HCV coinfection find no meaningful rebound [34]. In acute HCV, unconventional T-cell responses that include MAIT cells are readily detectable and can persist after viral clearance, underscoring a durable imprint of innate-like activation [35]. Overall, the literature supports a model in which pro-inflammatory cytokines (IL-12/IL-18) and chronic immune activation drive MAIT attrition and dysfunction in HCV, with incomplete recovery of function after DAA treatment.

## 3. Metabolic Dysfunction-Associated Steatotic Liver Disease and MAIT Cells

The recognition of the pathophysiologic relationship between obesity, type 2 diabetes, and metabolic syndrome consensus groups have redefined fatty liver disease to metabolic dysfunction-associated steatotic liver disease (MASLD) [36,37]. Across metabolic diseases and tissues, MAIT cells display a similar phenotype. Peripheral MAIT cells decrease significantly with obesity and type 2 diabetes [38,39]. In a similar trend, there is a reduction in circulating MAIT cells frequencies in patients with MASLD compared to healthy controls [40,41,42,43]. Despite reduction in numbers, MAIT cells in obesity (blood and adipose tissue) adopt a pro-inflammatory IL-17 expressing state and lose their regulatory tone (i.e., IL-10 expression) [39]. The IL-17 phenotype of MAIT cells is partially attenuated with antioxidants implicating dysfunction in mitochondrial ROS in this process [44]. MASLD, characterized by excess triglycerides and eventually scar (cirrhosis) deposition in the liver shows similar directional changes in MAIT as in adipose tissue. In MASLD, circulating MAITs are markedly decreased versus healthy controls, with an activated (CD25^+^ CD69^+^) and pro-inflammatory profile (IL-17^+^ and granzyme B^+^). Intrahepatic MAITs are also reduced, but redistribute from sinusoids into fibrotic septa, where they lie adjacent to α-SMA^+^ myofibroblasts and show signs of prolonged activation (PD-1^+^). In functional experiments, it was demonstrated that MAIT cells potentiate the release of pro-inflammatory molecules from macrophages (IL-6 and IL-8) and drive a pro-inflammatory program in hepatic myofibroblasts partially via IL-17 and TNF-α [40]. A 12-week pilot study in biopsy-proven MASLD demonstrated that dietary intervention reduced CD69 expression on circulating MAIT cells and improved steatosis scores [45]. Given, the rapidly evolving pharmacotherapy landscape of obesity (GLP-1 agonist) and MASLD (GLP-1 agonist and Resmetirom), it would be informative to test whether disease response is accompanied by the MAIT cell downregulation of activation and expression of pro-inflammatory cytokines, and do they track with changes in hepatic fibrosis. It is unknown whether lipotoxic stress in steatosis skews MAIT cells toward profibrogenic functions, or whether MAIT cells reinforce lipotoxic inflammation and injury, creating a feed-forward loop that accelerates fibrosis.

## 4. Alcohol-Associated Liver Disease and MAIT Cells

Alcohol-associated liver disease (ALD) encompasses at least two clinical tracks: a smoldering chronically inflamed course to cirrhosis, and a flare prone course of recurrent episodes of acute alcoholic hepatitis before cirrhosis. Circulating MAIT cells upregulate the markers of activation but are functionally impaired, and they appear to be numerically diminished in ALD, with a progressively more profound loss in alcoholic hepatitis patients (most significant inflammatory state) than in patients with alcoholic cirrhosis [46]. Circulating MAIT cells in alcoholic liver disease show enhanced expression of CD69, CD38, and HLA-DR compared to healthy controls, and this phenotype can be transferred by ALD plasma but not direct ethanol exposure in vitro [46]. Despite the upregulation of activation markers, MAIT cells in ALD display a blunted cytokine (IL-17) and cytotoxic (Granzyme B and CD107) response to *E. coli* challenge. These changes are accompanied by transcription factor expression defects in PLZF, RORC, EOMES and TBET signaling a loss in “innate-ness program” in MAIT cells in ALD. A possible mechanism for dysfunction is due to the altered microbial milieu in patients with ALD, as fecal extracts from ALD patients result in healthy PBMC MAIT cells acquiring similar defects to ALD MAIT cells in contrast to *E. coli* alone, ethanol, or plasma [46]. Single-cell transcriptomics on intrahepatic and peripheral immune cells in cirrhotic stage patients with ALD (vs. healthy controls), conversely showed an enrichment of Granzyme B expressing MAIT cells in the circulation and liver of diseased individuals [47]. Taken together, these studies underscore MAIT cell transcriptional and functional remodeling in alcohol-related liver disease. Differences in experimental endpoint (protein versus transcript abundance), tissue handling (fresh tissue versus dissociated single cells), patient cohorts (alcoholic hepatitis versus cirrhosis) could explain the divergent conclusions. These findings suggest that MAIT cells may be cytotoxic, likely primed at the transcript level, but functionally restrained due to the inflammatory milieu in ALD. However, paired transcriptional and protein/functional studies, integrated with microbiome mapping are needed to confirm these observations. Multiple studies have noted the contraction of MAIT cell in the periphery in the setting of alcohol use even without overt liver disease and MAIT cell numbers inversely correlate with disease duration [40,46,48,49]. Intrahepatic MAIT cells, meanwhile, are retained, possibly explained by expression of CXCR3, CX3CR1, CCR9, beta-7 integrin on MAIT cells, and increased expression of T cell chemoattractant CXCL10 in livers with alcohol-associated liver injury [46]. Abstinence from alcohol partially rescues MAIT cell frequencies and functional impairment at earlier stages of disease, but not at the cirrhotic stage, where MAIT cell loss becomes irreversible [48,49]. Since alcoholic hepatitis is marked by neutrophilic proliferation unlike alcoholic cirrhosis, a question remains whether cross talk with neutrophils or neutrophilic activation in alcoholic hepatitis drives the profound MAIT dysfunction and loss observed in alcoholic hepatitis compared to cirrhosis.

## 5. Biliary Tract Disease and MAIT Cells

The role of MAIT cells in biliary tract disease which encompasses primary sclerosing cholangitis (PSC), primary biliary cholangitis (PBC), and biliary atresia is gaining increasing attention. These diseases are characterized by a functional and/or structural impairment in bile flow, a process called cholestasis. This review section will delve into the current understanding of MAIT cell involvement in each of these distinct biliary pathologies.

PSC is a disease that involves a chronic injury of bile duct epithelial cells called cholangiocytes, leading to concentric areas of fibrosis around the bile ducts and secondary parenchymal damage. Effective medical therapy is lacking in PSC and the management centers on control of complications. MAIT cells are implicated in PSC for a number of reasons. Bile duct brushings obtained during endoscopic procedures from patients with PSC and non-PSC controls show that MAIT cells occupy a significantly higher proportion of the T cell population than in the periphery [50]. Similarly, it has been observed that MAIT cells preferentially localize to the peribiliary area [51]. Consistent with this peribiliary tropism, liver MAIT cells express receptors and integrins that promote the homing and retention of T cells around cholangiocytes, CXCR6, CCR6, and αEβ7 [52,53,54] as cholangiocytes express the corresponding chemokines CXCL16 and CCL20 [51,52,55,56]. These observations also raise the specter of the biliary microenvironment harboring MAIT cell antigens. Indeed, cholangiocytes induce MAIT secretion of IFN-γ in the presence of *E. Coli*, an effect that is attenuated with MR1 blockade suggesting cholangiocytes can function as non-canonical antigen-presenting cells (APCs) for MAIT cells [51]. Consistent with this finding, cholangiocytes and THP-1 cells (prototypical APC) pretreated with PSC patient bile activated MAIT cells in a TCR-mediated process [57]. A search for non-microbial host-derived ligands for MAIT cells to explain its preferred localization surrounding bile ducts revealed sulfated bile acids to be a potential ligand for MAIT cells [58]. Indeed, The single-cell RNA sequencing of human PBMCs were treated with 5-OP-RU (potent canonical stimulus) and sulfated bile acid cholic acid 7 sulfate (CA7S) demonstrated that CA7S selectively upregulated IL-7R [58], which is known to promote MAIT cell residency in the liver [59]. Interestingly, PSC patients show a decrease in the sulfating enzyme SULT2A1 expression compared to PBC and healthy controls indicating an impairment in bile acid detoxification [60]. MAIT cells are numerically reduced in the circulation and livers of PSC patients than healthy controls [51]. Circulating MAIT cells in PSC adopt a chronically activated phenotype with enhanced expression of CD69, PD-1, and CD39 compared to healthy controls. Despite this, PSC MAIT cells mounted a blunted CD107a, IFN-γ and TNF-α response to *E. Coli* (TCR mediated) stimulation whereas they maintained their response to TCR independent IL-12 and IL-18 stimulation [50].

PBC is an autoimmune, antibody-mediated cholestatic liver disease that predominantly targets small intrahepatic bile ducts and is generally responsive to medical treatment with ursodeoxycholic acid (UDCA). Multiple studies observe a reduction in circulating MAIT cells in PBC [61,62,63]. These MAIT cells exhibit higher expression of CD25 and CD69 as well as an enhanced capacity to secrete cytokines IL-17A, IFN-γ, and granzyme B. The reduction in MAIT cells is also associated with the upregulation of markers of apoptosis and of liver homing receptors CCR6 and CXCR6. Consistent with this notion, PBC livers expressed the corresponding cytokines to these receptors. Therefore, there are two possibilities (increased apoptosis and liver homing) to explain the peripheral reduction in MAIT cells in this disease. Whether MAIT cell frequencies in the liver increase or decrease in PBC is still equivocal. While one study which uses MR1 loaded tetramers to detect MAIT cells in liver sections noted an increase in MAIT abundance in the PBC liver [61], another study using flow cytometry CD161^+^ TCRα7.2^+^ gating strategy to identify MAIT cells noted the converse [62]. MAIT cells in PBC are responsive to both TCR and cytokine stimulation when compared to healthy controls. IL-7 (induced in hepatocytes by bile acid signaling) and IL-18 induced higher expression of cytokines (IFN-γ, IL-17A, TNF-α) in PBC patient peripheral MAIT cells than controls [61,63]. On the contrary, two other studies reported impaired MAIT cytokine response in PBC [62,64], implying differences in observations maybe due to patient cohorts, sample storage and handling. Patients who respond to UDCA therapy show the partial rescue of circulating MAIT cell abundance and attenuation in MAIT cell cytokine release, indicating MAIT cell activation and pro-inflammatory profile tracks with PBC disease activity. PBC is an autoantibody-driven autoimmune disease, implying a break in self-tolerance and pathogenic B cell responses. Whether MAIT cells amplify or restrain these processes is yet to be elucidated. Impairments in the function of regulatory T cells have been observed in PBC [65,66] and it is unknown whether MAIT cells play a role in this suppression [66]. In the context of other rheumatologic disease (Sjrodgren’s) MAIT cells express the B cell help cytokine IL-21 suggesting a role in pathogenically activating B cells [67]. Further studies are needed to investigate whether MAIT cells potentiate B cell activation in PBC.

Biliary atresia (BA) is a neonatal disease that progressively blocks the bile ducts causing cholestasis, inflammation and rapid progression to cirrhosis in infancy. Evidence on the role of MAIT cells in biliary atresia is sparse. However, a well-executed study employed patient-derived MAIT cells and complex multicellular models and found that BA patient MAIT cells exhibited a wound healing transcriptomic signature and MAIT-derived amphiregulin (AREG) promoted aberrant cholangiocyte proliferation, linking MAIT cells to fibroinflammatory progression in BA [68]. Using MAIT gene signature to qualitatively estimate MAIT abundance (MAIT high vs. low) in two published BA datasets, the authors showed that liver MAIT cell abundance correlated with fibrosis and cholangiocyte proliferation. In BA, circulating and liver MAIT cells showed signs of activation (HLA-DR and CD38) though cytokine responses were comparable. Intrahepatic MAIT cells showed a strong transcriptional activation of AREG, and in vitro studies confirmed its proliferative effect on cholangiocytes. In co-culture models MAIT cells stimulated cholangiocyte proliferation (hallmark of cholestatic injury) and the activation of hepatic myofibroblasts, suggesting a profibrogenic role of MAIT cells in BA. Further studies are needed to corroborate these findings and investigate if the wound healing properties of MAIT cells can be manipulated to treat BA.

## 6. Autoimmune Hepatitis and MAIT Cells

In autoimmune hepatitis (AIH), a disease characterized by the lymphocytic inflammation of the liver, MAIT cells show changes in abundance and function. Circulating MAIT cells drop, correlating with disease severity and do not recover despite prolonged immunosuppression and the normalization of liver biochemistries [14,69,70]. In fact, AIH patients have significantly more reduction in circulating MAIT cells compared to other extrahepatic rheumatologic diseases such as rheumatoid arthritis [70]. However, one subset of MAIT cells (CD4^+^) are enriched in patients with AIH while the CD8^+^ MAIT cell population shrinks [14]. Similarly to PSC and PBC, MAIT cells from AIH patients show the upregulation of HLA-DR, CD38, CD69 consistent with activation. Indeed, AIH MAIT cells demonstrate the high basal expression of TNF-α, IFN-γ, and IL-17 but mount a blunted response to PMA/ionomycin stimulation, suggesting an exhausted phenotype [70]. Consistently, functional assays also demonstrate that granzyme B and IFN-γ responses is impaired in AIH MAIT cells in response to TCR stimulation [69]. In contrast to circulating MAIT numbers, the intrahepatic MAIT cell population remains largely stable in AIH. The pathogenic role of MAIT cells in AIH is identified via in vitro studies that show hepatic myofibroblast proliferation and upregulation of inflammatory genes when co-cultured with MAIT cells [14]. Although the majority of circulating human MAIT cells are CD8^+^, CD4^+^ MAIT cells remain an elusive subset and future studies should focus on distinguishing the cytotoxic, tissue homing, and wound healing functions of CD4^+^ MAIT cells compared to their CD8^+^ or CD4^−^ CD8^−^ counterparts (Figure 1).

## 7. Hepatocellular Carcinoma and MAIT Cells

Hepatocellular carcinoma (HCC), the most common primary liver malignancy, and a leading cause of cancer-related mortality, typically emerges on a background of chronic inflammation and fibrosis. In a multi cohort analysis, tumor-infiltrating MAIT cells were found to be scarce, upregulating TIM3 and CTLA-4 with attenuated Granzyme B and perforin response suggesting decreased cytotoxic potential [71,72]. Conversely, MAIT cells increased tumor promoting cytokine IL-8, which is known to promote angiogenesis and invasion in HCC [73]. MAIT density in the tumors correlated with worse survival arguing for a pathogenic role of these cells in HCC. Conversely, through high parameter spatial (CODEX) and single-cell RNA sequencing approaches on human HCC samples, a protective role of MAIT cells emerged, where PDL-1^+^ CD163^+^ tumor-associated macrophages (TAM) exerted a suppressive effect on MAIT cells via cell to cell contact and PDL-1/PD-1 signaling. An alternative mechanism of MAIT cell dysfunction by metabolic stress is proposed by a recent study which found that polyunsaturated fatty acids drove lipid peroxidation and ferroptosis in MAIT cells [74].

## 8. Future Directions

Across liver diseases of diverse etiology, viral hepatitis, MASLD/MASH, autoimmune liver diseases, cholangiopathies, and HCC, a consistent but poorly understood observation, is a drop in circulating MAIT cells. This fall has been variously attributed to activation-induced cell death and to redistribution toward a diseased liver; yet, intrahepatic accumulation is not consistently seen in liver disease, even when MAIT cells are implicated in pathology. Key questions therefore remain: does the decline in blood MAITs contribute to pathogenesis, is it a bystander of systemic inflammation, or do two largely independent processes coexist, loss from the circulation and context-dependent retention within the liver? Regardless, the field’s emphasis should shift to MAIT cells at the site of injury, particularly within peri-sinusoidal and peribiliary niches where they likely exert the greatest influence. Because access to human liver tissue is constrained by obvious ethical and logistical limits (and often yields insufficient MAIT numbers for deep assays), investigators can leverage spatial transcriptomics and single-cell approaches (e.g., scRNA/CITE-seq with TCR sequencing, spatial RNA/protein mapping) to define MAIT states and interactions in situ without requiring large-tissue samples. The most informative designs would include serial sampling of the same patient for example, biopsies across disease stages or pre/post therapy to track phenotypic and functional shifts in hepatic MAIT cells over time. Findings from the liver can then be tested and mirrored in the periphery by (i) conditioning healthy-donor MAIT cells with disease-specific cues, e.g., IL-12/IL-18, bile acids, conjugated bilirubin or supernatants from hepatocytes treated with lipotoxic stimuli to mimic MASH to see whether hepatic MAIT programs can be induced, and/or (ii) profiling patient PBMC-derived MAITs as a feasible surrogate readout. Together, these strategies can disentangle whether blood MAIT loss is causal, correlative, or compartment-specific, while anchoring conclusions in the anatomic context where MAIT cells act. Another relative unchartered territory is the role of MR1 expression in MAIT cell homing dynamics in disease. MR1 expression is inducible by inflammation [40,75,76] and appears increased on liver epithelia and myeloid cells in several liver diseases [19,41,46]. A key unresolved question is whether this MR1 upregulation directly retains MAIT cells at injury sites via ongoing antigen-dependent engagement (“antigenic tethering”), or whether MR1 expression simply tracks with a cytokine/chemokine milieu (e.g., IL-7, IL-12/IL-18; CXCL16/CXCR6, CCL20/CCR6, CXCL12/CXCR4) that independently recruits and anchors MAIT cells. Disentangling these possibilities will require spatially resolved MR1 quantification and perturbation studies (MR1 blockade/ligand depletion vs. chemokine–axis inhibition) in human liver tissue, organoids, or ex vivo precision-cut slices. Because murine MAIT cells are scarce and disease modeling is shifting beyond rodents, perturbation studies should move past planar co-culture toward human organoids, assembloids, and microfluidic “organ-on-chip” systems. Gut-derived organoids have already been used in the MAIT field [77], but such classical Matrigel-based organoids still fall short to model liver disease in vitro as they incompletely capture the multicellular complexity, lack matrix mechanics that drive fibrogenesis, and miss higher-order tissue architecture. Recently, a mouse periportal liver assembloid combining portal mesenchyme, cholangiocytes, and hepatocytes was developed [78]. The periportal assembloid demonstrated functional bile flow offering a compelling scaffold for disease modeling, yet it lacks resident immune cells. Future studies should refine this platform using human cells and incorporate MAIT cells and enable controlled studies of MAIT-epithelial and MAIT-myofibroblast crosstalk with tunable MR1 ligands, cytokines, and bile acids. Finally, microfluidic devices that co-perfuse blood-side and bile-side channels and support multi-lineage co-culture can deliver physiologic shear, gradients, and flow, allowing a direct interrogation of MAIT behavior under injury-mimicking conditions. Collectively, these human-centric systems will move the field from descriptive snapshots in tissue to causal, perturbation-based biology that links MAIT cell biological mechanisms to disease phenotype.

## 9. Evolving Framework of MAIT Cell Investigations

### 9.1. Conceptual

We return to the basic principles of the liver comprising a true mucosal surface in the form of bile ducts, and functional mucosa in terms of hepatocytes coming into direct contact with portal blood in the sinusoids. Each of these mucosal surfaces are surrounded by their respective mucosal-adjacent niches. The bile ducts are surrounded by the periductal niche, and the hepatic sinusoids are surrounded by the perisinusoidal niche. MAIT cells have been detected in bile duct brushings obtained during invasive endoscopic procedures to examine the biliary tree (e.g., endoscopic retrograde cholangiography (ERC) which directly samples the biliary mucosa [50]. These biliary MAIT cells can be considered to be truly mucosal as they sample antigens in bile and stand as sentinels in preventing microbial injury from bile sources. However, MAIT cells have also been detected in the periductal niche where they are unlikely to encounter bile-derived antigens and can be considered periductal MAIT cells.

**Figure 2:**
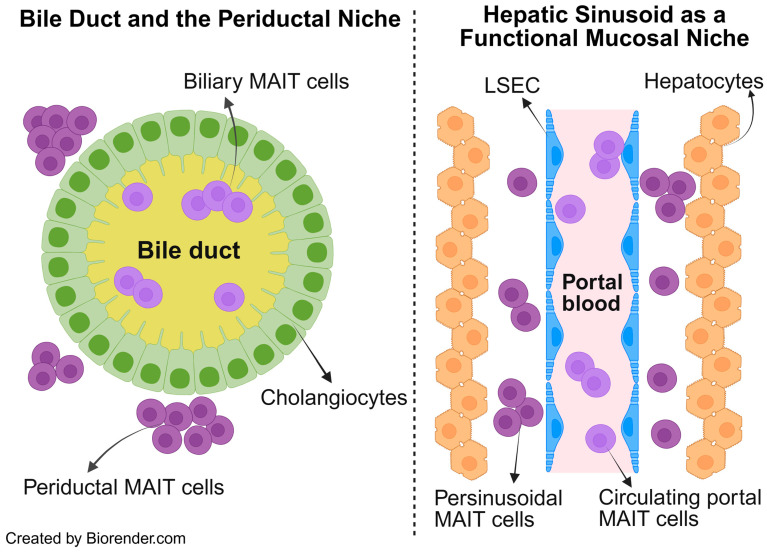
Conceptual framework of intrahepatic MAIT cells. The liver’s unique architecture comprises a true mucosal surface which are bile ducts lined by cholangiocytes (**left**) and a functional mucosa, which is the hepatic sinusoid lined by fenestrated LSEC and surrounded by hepatocytes (**right**). Intrahepatic MAIT cells are conceptualized into four distinct populations based on which niche they occupy within this specialized liver architecture. Biliary, periductal, perisinusoidal, and circulating portal blood MAIT cells. Abbrv. LSEC, liver sinusoidal endothelial cell.

Likewise, the perisinusoidal MAIT cells are positioned in a functional mucosal niche between hepatocytes and the hepatic sinusoids lined by fenestrated liver sinusoidal endothelial cells (LSECs), where they are exposed to portal blood draining the intestines. This portal blood not only carries microbial/nutrition products but also a circulating pool of MAIT cells that continuously recirculates through the liver. These intravascular MAIT cells can transiently survey the sinusoidal lumen or re-enter the systemic circulation after being imprinted by the hepatic microenvironment. Building on these basics, we propose that intrahepatic MAIT cells be conceptualized as biliary, periductal, perisinusoidal, and circulating portal blood populations, as illustrated in Figure 2.

Most of the “action” or immune hub in chronic fibrotic liver diseases happens in the periductal (biliary tract disease) or perisinusoidal space. It is in this larger niche of the periductal or perisinusoidal space that MAIT cells are likely to perform in intercellular communication with other immune cells to create a pro-inflammatory and potentially profibrogenic milieu. It is also where MAIT cells (and other immune cells) encounter the hepatic myofibroblasts which are the central orchestrators of fibrogenesis. Therefore, it is highly conceivable that the MAIT cells which display a wound healing response would be localized to the periductal or perisinusoidal niche. The key unresolved questions are whether MAIT cells within these specialized niches are merely recruited responders shaped by the pre-existing fibroinflammatory microenvironment, or whether they actively orchestrate, amplify, and sustain this environment in ways that perpetuate chronic liver injury and fibrosis.

### 9.2. Technical

A complementary technical framework is needed to dissect these distinct intrahepatic MAIT cell niches. Key advances are likely to come from integrating high-resolution spatial technologies with targeted MAIT cell profiling. Biliary MAIT cells obtained from bile duct brushings should be subjected to detailed characterization using single-cell transcriptomic and proteomic approaches, enabling the direct comparison of biliary MAIT cells with their counterparts in blood and liver tissue. In parallel, ex vivo human liver studies will benefit from robust in situ methodologies. These approaches will require well-validated antibody panels that reliably identify MAIT cells by conventional immunofluorescence, supplemented by RNA in situ hybridization to enhance specificity. However, these conventional techniques remain inherently limited in molecular depth and multiplexing. The most informative strategy will likely involve single-cell spatial transcriptomic platforms, with MAIT cell identifying markers incorporated into custom probe panels. Such technologies would allow the simultaneous mapping of MAIT cells at mucosal surfaces versus the periductal/periportal compartment, while decoding their signaling pathways, ligand–receptor interactions, and cellular neighborhoods. In doing so, they could reveal how MAIT cells are positioned within, and potentially shape, the inflammatory and fibrogenic microenvironments that characterize chronic liver disease.

## 10. Conclusions

MAIT cells occupy strategically positioned mucosal and mucosal-adjacent niches in the liver where they can not only contribute to classic antimicrobial defense but also have the potential to amplify the immune and fibrogenic processes in disease. Deciphering whether MAIT cells are bystanders of dysregulated mucosal and mucosal-adjacent environments or whether they are the drivers of pathogenic processes will be critical in revealing their value as biomarkers or targets for therapies of chronic liver disease.

**Table 1:** Summary of the reported alterations in MAIT cell abundance and function across major liver diseases described in this review with supporting studies cited by reference numbers.

Liver Disease	MAIT Functional Changes (Highlights)	References
Hepatitis B (HBV)	- Reduced MAIT abundance in blood and liver; chronic activation/exhaustion phenotype - Functional changes: Dampened cytokine expression; MR1-dependent degranulation/cytotoxicity against HBV-transfected hepatocytes	[17,18,19,20,21,24]
Hepatitis C (HCV)	- Reduced circulating and liver MAIT frequencies with heightened activation/exhaustion signatures - Functional changes: Blunted IFN-γ/TNF-α responses and altered degranulation; dysfunction often persists despite successful HCV treatment	[25,26,27,28,29,30,31,32,33,34,35]
MASLD/MASH	- Decreased circulating and hepatic MAIT frequencies; hepatic MAIT cells redistribute into fibrotic septa near myofibroblasts; prolonged activation phenotype - Functional changes: Enhanced expression of IL-17A; promote pro-inflammatory programs in hepatic macrophages and myofibroblasts	[40,41,42,43,44,45]
Alcohol-associated liver disease (ALD)	- Reduced in circulation (most profound in alcoholic hepatitis) but retained in the liver; chronic activation signatures - Functional changes: Impaired antibacterial effector function to *E. coli* stimulation (reduced IL-17A and cytotoxic readouts) with transcription factor defects (PLZF, RORC, EOMES, TBET); functional impairment partially restored with alcohol abstinence in early stage of disease.	[46,47,48,49]
Primary sclerosing cholangitis (PSC)	- Enrichment of MAIT cells in bile duct brushings and peribiliary localization; reduced abundance in blood; chronically activated phenotype - Functional changes: Blunted TCR-dependent responses to *E. coli* (e.g., CD107a, IFN-γ, TNF-α) but preserved cytokine-driven (IL-12/IL-18) responses	[50,51]
Primary biliary cholangitis (PBC)	- Reduced circulating MAIT frequencies with increased penchant for apoptosis and/or liver homing (CCR6, CXCR6) - Functional changes: Enhanced cytokine/cytotoxic mediator capacity (IL-17A, IFN-γ, granzyme B) alongside cohort-dependent findings of impaired responses in some studies	[14,61,62,63]
Biliary atresia (BA)	- Significant reduction in MAIT cell frequencies in blood and liver; wound-healing transcriptional program - Functional changes: MAIT cells promote aberrant proliferation of cholangiocytes and activation of hepatic myofibroblasts	[65]
Autoimmune hepatitis (AIH)	- Decreased frequencies in the circulation which do not recover despite treatment; activation markers - Functional changes: High basal cytokine expression but show impaired granzyme B/IFN-γ to TCR stimulation (exhaustion-like); promote hepatic myofibroblast proliferation in vitro	[14,66,67]

## Data Availability

No new data were created or analyzed in this study.
